# The Prospects and Challenges of Live Attenuated Vaccines Against African Swine Fever Virus in Vietnam

**DOI:** 10.3390/vaccines14030284

**Published:** 2026-03-23

**Authors:** Tram Thi Ngoc Ngo, Taehwan Oh, Duy Tien Do

**Affiliations:** 1 Department of Infectious Diseases and Veterinary Public Health, Faculty of Animal Science and Veterinary Medicine, Nong Lam University HCMC, Ho Chi Minh City 70000, Vietnam; tramngo.vsrt@gmail.com; 2 The Animal Biomedical Research Laboratories, Nong Lam University HCMC, Ho Chi Minh City 70000, Vietnam; 3 Department of Microbiology, College of Medicine, Dankook University, Cheonan 31116, Republic of Korea; ohth@dankook.ac.kr

**Keywords:** African swine fever virus, live attenuated vaccine, Vietnam, molecular epidemiology, vaccine efficacy, viral evolution

## Abstract

African swine fever (ASF) is a contagious viral disease that causes severe economic losses in the global swine industry. Since its introduction to Vietnam in 2019, ASFV has evolved rapidly, with genotype II strains dominating initially and recombinant I/II variants emerging by 2023. Live attenuated vaccines (LAVs) have been developed and commercialized in Vietnam, including ASFV-G-ΔI177L, ASFV-G-ΔI177L/ΔLVR, and ASFV-G-ΔMGF, which confer homologous immune protection. Despite this, LAVs face challenges related to genetic stability, impossible protection against emerging recombinant strains, potential reversion to virulence, viral shedding, and safety in pregnant sows. ASFV’s ongoing evolution underscores the need for continuous genomic surveillance, evaluation of cross-protective efficacy, and implementation of biosecurity and DIVA strategies focused more on evaluating vaccine efficacy than safety. This review summarizes the current molecular epidemiology of ASFV in Vietnam after vaccines were licensed for use, the development and performance of commercial LAVs, and the practical challenges of their application in endemic settings, and provides insights for informed vaccine deployment and integrated ASF control strategies in rapidly evolving viral landscapes.

## 1. Introduction

African swine fever (ASF) is a contagious transboundary disease of pigs that poses a major threat to the global swine industry and results in substantial socioeconomic losses and environmental consequences. The disease is caused by the *African Swine Fever Virus* (ASFV), a large double-stranded DNA virus belonging to the genus Asfivirus, family *Asfarviridae*. The disease is characterized by high fever and clinical signs related to hemorrhage, with mortality rates that approach 100% in infected pigs depending on the virulence of the virus. Furthermore, no specific treatment is available currently [1,2]. Since its entry into China in 2018, African Swine Fever (ASF) has spread rapidly throughout Southeast Asia, including Vietnam, where it has led to the loss of millions of pigs of all ages and disrupted the pork supply chain, a crucial component of national food security [3,4]. Vietnam recorded its first case of ASF in early 2019, and although many drastic control measures were implemented, the virus has now become endemic in many localities [5]. Molecular epidemiological studies indicate that most circulating virus strains in Vietnam belong to genotype II, which is related closely to the highly virulent Georgia 2007/1 strain that caused the widespread ASF pandemic in the Eurasian region [6,7]. Nevertheless, genetic shifts are occurring rapidly, and increasingly diverse strains of the ASF virus are emerging in the field, including numerous mutations and deletions in genes related to virulence and immune evasion, such as *EP402R/CD2v* and genes belonging to the *MGF360/505* family [8,9,10]. 

In recent years, live attenuated vaccines (LAVs) have emerged as one of the most promising strategies to control ASF, largely because of their capacity to elicit balanced humoral and cell-mediated immune protection [11,12,13]. Several LAV candidates developed from attenuated ASFV strains attributable to gene deletions or natural attenuation have shown protective efficacy under experimental conditions, including the *DP148R*, *MGF360/505*, and *CD2v* gene deletion mutations [14,15]. ASF LAVs developed in Vietnam have progressed to field trials and received commercial authorization, which marks a significant milestone in the global effort to mitigate ASF [16]. However, several studies have highlighted issues related to these vaccines’ genetic stability, safety, and virulence, as well as their efficacy under diverse field conditions [8,17,18,19]. To our knowledge, this is the first review focusing on Vietnam’s post-licensure LAV deployment together with contemporaneous genomic shifts, including the expansion of recombinant I/II strains. 

## 2. Method

### 2.1. Literature Search

A narrative structured review was conducted following key principles of the PRISMA (Preferred Reporting Items for Systematic Reviews and Meta-Analyses) guidelines to enhance transparency and reproducibility [20], although a formal systematic review and meta-analysis were not performed.

A comprehensive literature search was performed across major scientific databases, including PubMed, Web of Science, and Scopus, supplemented by national surveillance reports and official technical documents from veterinary authorities. The search strategy combined relevant keywords, including: “African swine fever virus” OR “ASFV” AND “genomic variation” OR “mutation” OR “multigene family (MGF)” OR “recombination” AND “vaccine” OR “attenuation” OR “live attenuated vaccine”. Reports and grey literature available from the Department of Animal Health (DAH), the Food and Agriculture Organization of the United Nations (FAO), and the World Organization for Animal Health (WOAH), and the United States Department of Agriculture (USDA) were also used in this analysis.

### 2.2. Study Selection

Studies were selected according to the following inclusion criteria: (i) original research or review articles addressing ASFV genomic variation; (ii) studies reporting mutations, MGF deletions, or recombination events; and (iii) relevance to vaccine development, attenuation mechanisms, or post-vaccination observations. Exclusion criteria included studies lacking genomic or vaccine-related data, non-peer-reviewed sources (except official reports), and articles with insufficient methodological detail. Finally, the eligible studies were analyzed qualitatively, and available data were synthesized to explore the relationships between ASFV genetic variation and vaccine design, attenuation strategies, and post-vaccination observations.

A total of 1873 articles were initially identified. After removal of duplicates and screening based on titles and abstracts, 1593 articles were excluded, resulting in 280 articles included in the full-text analysis. Based on the research criteria, 102 articles were selected for analysis. Additionally, the grey literature comprised two conference presentations, one technical report, two in-press conference proceedings, and one document issued by the Department of Animal Health (DAH).

## 3. Live Attenuated Vaccines: Current Global Progress

Despite decades of research, developing a safe and effective ASFV vaccine remains challenging [21]. Historical research has seen diverse approaches, including inactivated vaccines, subunit vaccines, DNA, and viral vectors [22]. Inactivated vaccines (that use heat or chemicals) and subunit vaccines, while considered highly safe, fail consistently to protect against virulent ASFV. These vaccine platforms primarily induce humoral immune protection without eliciting sufficiently robust cellular immune protection. This imbalance between humoral and cellular immunity is likely a key factor contributing to their limited protective efficacy [23,24,25]. Similarly, while they stimulate antibody production, DNA vaccines and viral vectors often offer inconsistent or limited protection [26,27,28,29,30]. Even mRNA technology, which has been developed successfully for other diseases, remains in the experimental stage for ASFV because of immunological challenges [31,32]. Efforts to develop DNA vaccines have made promising progress, improving cell-mediated immune protection and enhancing protection in challenge tests [33,34].

**Figure 1:**
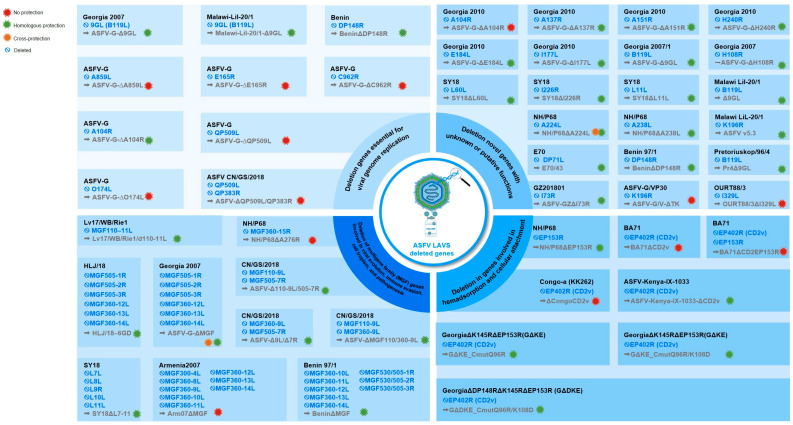
Schematic classification of genes deleted in current LAV candidates against ASFV. Colors denote protection level: red, none; green, homologous; orange, cross-protection.

In this context, live attenuated vaccines (LAVs) are currently considered the most promising strategy because of their ability to stimulate balanced and sustained immune protection [21,22]. LAV candidates are developed in three main ways: (1) naturally attenuated; (2) attenuated through cell culture passage; and (3) attenuated through genetic engineering (gene deletion) [35,36,37,38]. Low-virulence ASFV strains isolated in the field show potential in the development of vaccines against ASFV. Results from experiments conducted on naturally occurring low-virulence strains show protection rates ranging from 33–100%, but animals develop chronic clinical conditions after vaccination and the safety of naturally occurring low-virulence vaccine candidates is not guaranteed [39,40]. Attenuated ASFV vaccine candidates obtained through multiple passages in cells show a decrease in viral virulence and are safe for vaccination in pigs. In some cases, the serial passage process may be accompanied by a decrease in immunogenicity, leading to less than desired protective efficacy [41]. However, recent research by Truong et al., 2023, shows that the attenuated virus strain obtained through multiple passages (VNUA-ASFV-LAVL2) still maintains good immunogenicity, is safe for vaccinating pigs, and provides effective protection against challenge with highly virulent virus strains [42]. In fact, gene-deleting strategies are dominating 21st century research [22] as they offer strong homologous protection and even partial cross-protection [11,12,13,43]. Generally, attenuation of ASFV is achieved through targeted deletion of virulence-related genes that regulate viral replication, immune evasion, and dissemination within the host. These genes can be categorized into four main functional groups [38] (Figure 1). A key indicator of this approach is the commercial approval in Vietnam of three genotype II-based deleted LAVs (ASFV-G-ΔI177L, ASFV-G-ΔMGF, and ASFV-G-ΔI177L/ΔLVR) [16,44,45].

### 3.1. Gene Deletions Essential for Viral Genome Replication

This group of genes encodes proteins that are involved directly in viral DNA synthesis, the formation of the replication complex, and virion assembly. These genes’ partial or complete deletion reduces viral replication efficiency and limits virus dissemination, thereby leading to attenuation of virulence. For example, deletion of the *9GL (B119L)* or *DP148R* genes has been shown to disrupt virion maturation, inhibit viral growth in vitro, and result in attenuated virulence in vivo [14,15]. Specifically, the *DP148R* gene, located near the right terminal region of the ASFV genome, plays a role in modulating virulence in macrophages. Deletion of DP148R in the Benin genotype I strain (BeninΔDP148R) has been demonstrated to confer complete protection against homologous challenge. Further, the product of the 9GL gene is involved in virion assembly, and deletion of this gene in the Georgia 2007 strain (ASFV-G-Δ9GL) results in reduced virulence; however, protective efficacy in pigs is incomplete unless combined with additional gene deletions.

### 3.2. Multigene Family (MGF) Gene Deletions

*Multigene families (MGFs)* are a hallmark genetic characteristic of ASFV and constitute approximately 30% of the viral genome [46]. MGF genes are categorized into five groups according to the average length of their protein products: *MGF100*; *MGF110*; *MGF300*; *MGF360*, and *MGF505* [47]. These gene clusters are primarily localized in two highly variable regions at the terminal end of the ASFV genome, referred to as the left variable region (LVR) and the right variable region (RVR) [46]. The number of MGF genes differs markedly among ASFV strains. In particular, naturally attenuated or cell culture-adapted strains tend to contain fewer MGF genes compared with highly virulent strains, indicating that some MGF genes may be dispensable for viral replication [48,49]. During viral entry and replication, proteins encoded by *MGFs* play a critical role in modulating the host’s innate immune protection, facilitating immune evasion and supporting efficient viral replication in the primary target cells: macrophages [50,51]. Previous studies have demonstrated that MGF genes can support viral replication in tick cells [52] and suppress type I interferon (IFN) production, thereby enabling the virus to evade the host’s innate immune protection [50]. Several members of the *MGF*, particularly *MGF360 and MGF505*, have been shown to inhibit type I interferon (IFN-I) production by interfering with key transcription factors such as IRF3, IRF7, and NF-κB, leading to reduced expression of IFN-β and interferon-stimulated genes [53,54,55].

### 3.3. Gene Deletions Involved in Hemadsorption and Cellular Attachment

Hemadsorption (HAD) is a characteristic phenomenon in which red blood cells adhere to the surface of ASFV-infected cells and is commonly observed in the majority of pathogenic field isolates. However, some naturally low-virulence ASFV isolates do not exhibit this phenomenon [56,57,58]. Structural and membrane-associated genes such as *EP402R* (CD2v) and *EP153R* (C-type lectin) regulate hemadsorption and facilitate viral dissemination through interactions with red blood cells and cell-to-cell spread [59]. Deletion of the CD2v gene reduces virion adsorption and limits viral spread in vivo, thereby contributing to reduced virulence, while it maintains the ability to induce some immune protection [60].

### 3.4. Novel Gene Deletions with Unknown or Putative Functions

Recent studies have identified several novel open reading frames (ORFs), among which, *I177L* plays a role in ASFV virulence through mechanisms that are not yet fully understood. Deletion of the *I177L* gene significantly reduced viral virulence without affecting host immunogenicity, suggesting that this gene is a plausible target for viral weakening [12]. 

In addition to *I177L*, a number of other conserved ASFV genes with either partially defined or predicted functions have been implicated in viral virulence, immune regulation, and replication. Notably, *NL (DP71L)*, *UK (DP96R)*, *and 9GL (B119L)* are highly conserved among genotype II isolates and are considered critical virulence factors [61,62]. *NL/DP71L* interacts with the host’s protein phosphatase 1 (PP1) to inhibit the phosphorylation of eIF2α, thereby preventing cellular translational repression [63]. *UK/DP96R* is involved in the efficient replication of the virus within macrophages [64], while *9GL/B119L* plays a vital role in virion maturation; deletion of this gene results in a marked reduction in virulence while it maintains immunogenicity, making it a potential target for the design of attenuated vaccines [65,66]. The second group comprises less well-characterized, but highly conserved ORFs, including *A104R*, *K196R*, *QP383R*, *QP509L*, and several other genes involved in viral replication, which have been reported to play roles in viral replication efficiency and structural integrity [67]. These genes encode proteins predicted to be involved in DNA replication, RNA modification, or viral inclusion body assembly; although their specific molecular functions are not yet elucidated fully, reverse genetic studies suggest that disrupting these ORFs reduces replication capacity or causes aberrations in virion formation [68,69].

Another group of ASFV genes is involved primarily in the regulation of the host’s immune protection and immune evasion. *A137R*, *A224L*, *A238L*, and *DP71L* encode proteins that participate in the inhibition of apoptosis, suppression of NF-κB signaling pathways, and modulation of host inflammatory responses [70]. Among these, *A224L* is an inhibitor of apoptosis protein (IAP), a protein that suppresses caspase-3 activity and promotes anti-apoptotic mechanisms in infected cells [71]. *A238L* modulates host inflammatory responses strongly by suppressing TNF-α and COX-2 expression through inhibition of NF-κB and AP-1 activation, and also interferes with T-cell signaling [72,73]. Recent evidence indicates that *A137R* also antagonizes the cGAS–STING pathway, which results in reduced type I interferon (IFN-β) production and subsequent impairment of innate immune protection [70,74].

## 4. The Rapid Shift in Molecular Characteristics of ASFV in Vietnam

ASF was first reported in Vietnam in 2019 and spread rapidly nationwide, causing severe economic losses and threatening food security. During the first year of the outbreak, the number of pig farms declined sharply and led to a reduction in pork production of up to 13.8% [75]. These adverse effects demonstrate that ASF represents not only a major animal health threat, but also a disease with far-reaching consequences across the entire pork production value chain. Vietnam has the 5th largest pig herd in the world and a massive pork food chain, which provides a comprehensive epidemiological landscape and valuable lessons for understanding ASFV evolution and developing effective disease prevention and control strategies.

From 2019–2021, studies identified all ASFV isolates circulating in Vietnam as belonging to genotype II, serotype VIII, which shows high genetic similarity with the Georgia/2007/1 reference strain and closely related strains circulating in China [76,77]. In the early phase of the epidemic, the virus exhibited high genetic stability, and no novel variants were reported. However, since 2021, several studies have identified the emergence of variants in the intergenic region (IGR) between I73R and I329L, including IGR II, IGR III, and notably IGR IV, which was reported for the first time in Vietnam [78]. These variants’ origin, whether attributable to local viral evolution or external introduction, has yet to be elucidated. Comparative genomic analyses of ASFV isolates from the 2019 and 2022 outbreaks revealed genetic variations in multiple ORFs. Although they remain within the IGR II classification, these strains showed close phylogenetic relations with ASFV lineages circulating in Europe and China [79]. Analyses conducted in southern Vietnam continued to reveal increasing genetic diversity, including the detection of novel variant strains in the central variable region (CVR) and IGR [80,81]. Importantly, in 2023, a report documented the emergence of a recombinant ASFV strain of genotype I/II in northern Vietnam, characterized by multiple recombination breakpoints, and found genetic features that resembled strains identified in China. Subsequent studies have confirmed that existing LAVs available in Vietnam fail to confer effective protection against this variant [82,83]. 

ASFV genotype II was first identified in China in 2018 and spread rapidly to at least 15 Asian countries [4]. By 2021, genotype I was also reported in China [84], and resulted in co-circulation of genotypes I and II and the subsequent emergence of recombinant strains. These recombinant strains were initially detected in China and appeared later in Vietnam in 2023 [83,85]. Unlike China, only genotype II and the I/II recombinant strains have been reported in Vietnam to date, with no evidence of genotype I circulation [83].

The emergence of recombinant ASFV (rASFV I/II) poses significant challenges for diagnostic and epidemiological surveillance, as traditional molecular markers such as *B646L*, *B602L*, *EP402R*, and the I73R–I329L intergenic region (IGR) become less effective for accurate genotyping of novel strains because of the mosaic nature of the genetic material derived from different genotypes [62,86,87]. Lee et al.’s (2024) study demonstrated that recombinant ASFV (rASFV I/II) isolates from Vietnam exhibit hemadsorption (HAD-positive) although they possess genotype I at the *B646L* locus [88]. Whole-genome analyses identified 19 recombination breakpoints, with nucleotide identities of 99.86–99.98% compared to Chinese strains. Variations in *C962R*, *I329L*, and *MGF505-11L* distinguish the Vietnamese strains from those in China, while mutations in *C122R* and *NP1450L* differentiate them from the parental strains. These isolates are classified as IGR II based upon the I73R–I329L intergenic region [88]

Post-commercialization surveillance following LAV deployment in Vietnam revealed increased ASFV genetic diversity, including in non-vaccinated herds. Analysis of 298 samples from 11 provinces identified co-circulation of vaccine-like strains, spillover variants, and emerging wild-type microvariants with non-synonymous SNPs and large deletions in the *EP153R–EP402R* region [89]. A recurrent 15-nt insertion in the E183L gene that resulted in a five-amino acid insertion (R–P–A–T–N) in p54 was consistently detected and represents a molecular signature of Vietnamese ASFV strains (Thanh et al., 2026 [89]). Concurrent analysis of 359 samples from 26 provinces revealed rapid expansion of rASFV I/II. Although p72 typing indicated genotype II predominance (73.1%) during 2023–2024, all genotype I isolates carried genotype II-derived p54 and *CD2v* genes. The prevalence of rASFV I/II increased from 14.1% in 2023 to 48.0% in 2025. Monthly analysis revealed a sharp rise from 12.5–22.2% in January–March to 59.5–77.4% in June–July, indicating rapid dissemination and potential epidemiological dominance [90]. 

**Figure 2:**
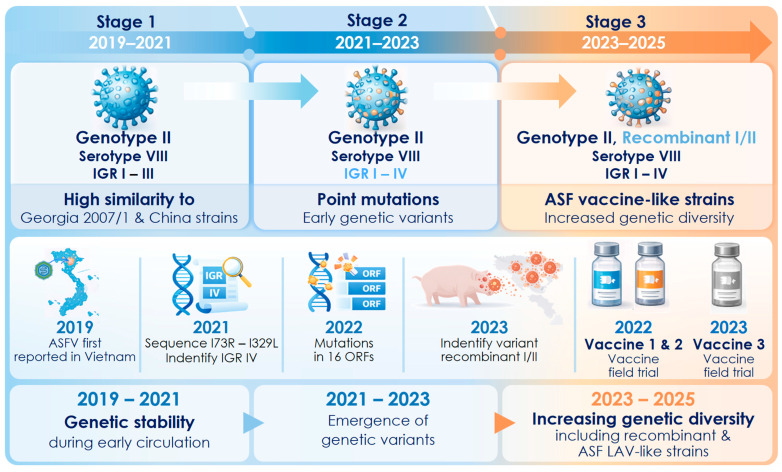
Temporal genetic shifts in ASFV in Vietnam during 2019–2025.

In particular, aspects of surveillance data from the Department of Animal Health, Vietnam [91] indicate that among 127 ASFV samples sequenced, 63.78% (81/127) were genotype II, while 36.22% (46/127) exhibited recombination between *B646L* (genotype I) and *E183L* (genotype II). By 2025, the prevalence of recombinant strains increased significantly to 44.74% (17/38), in which, genotype II accounted for 55.26% (21/38) [91]. Further, recent studies have reported the vaccine-like ASFV-G-∆MGF [8,18], which is capable of inducing clinical disease, as well as highly virulent rASFV I/II variants [92] (Figure 2).

In 2022, making a significant step forward in efforts to control ASF, Vietnam began commercializing the ASFV vaccines and introduced two products: ASFV-G-ΔI177L (with deletion of the I177L gene from the ASFV Georgia strain) and ASFV-G-ΔMGF strains (deletion of six multigene family MGF360 and MGF505) [93]. Both vaccine candidates, ASFV-G-ΔI177L and ASFV-G-ΔI177L/ΔLVR, were developed using the ASFV Georgia 2007/1 backbone obtained through collaborative research and technology transfer involving the United States Department of Agriculture (USDA). 

In early 2025, ASFV-G-ΔI177L/ΔLVR (deletion of the I177L gene and the Left Variable Region (LVR), which completely deletes 9 genes from ASFV Georgia strain) became the third ASFV vaccine strain to be licensed commercially in Vietnam [45]. Experimental studies conducted under both laboratory and field conditions indicate that these vaccines confer a measurable level of protective immunity [16,94,95,96,97]. However, at the same time, several international studies have raised warnings about the potential risks of reversion to virulence and genetic recombination of live attenuated ASFV vaccines, highlighting the need for strict field surveillance [98]. The commercial deployment of these vaccines marks a pivotal advancement in ASF control in Vietnam. Nevertheless, together with their benefits in disease prevention, vaccine viruses exert selective pressure in the field and may potentially influence viral evolution. Consequently, while many countries remain in the experimental stage, Vietnam has entered large-scale vaccination, which necessitates rigorous and continuous genetic surveillance of circulating viruses, as well as comprehensive evaluation of vaccine efficacy, to ensure sustainable epidemiological safety.

## 5. Current ASF Vaccines Licensed in Vietnam

Today, three genotype II live attenuated ASFV vaccines with specific gene deletions (ASFV-G-ΔI177L, ASFV-G-ΔI177L/ΔLVR, and ASFV-G-ΔMGF) have been licensed commercially in Vietnam, which marks a major step forward in the fight against ASF and contributes to promoting the recovery and sustainable development of the domestic pig farming industry. 

### 5.1. ASFV-G-ΔI177L

The *I177L* gene encodes a highly conserved protein of 161–177 amino acids, depending upon the ASFV isolate, and has been classified as a late gene in the viral replication cycle [12]. Recent evidence indicates that *I177L* acts as an immunomodulatory protein that regulates ASFV-induced inflammation by activating NF-κB signaling and the NLRP3 inflammasome concomitantly, thereby contributing to pathogenesis [99]. The ASFV-ΔI177L strain has been shown to confer robust protection against challenge with highly virulent ASFV following intramuscular (IM) [12,16] or oronasal (ON) administration [100]. Protective efficacy was consistently demonstrated against multiple ASFV strains, including ASFV-G and ASFV CN/GS/2018. Notably, administration at high doses (up to 10^6^ HAD_50_) or repeated vaccination did not result in abnormal clinical signs in 6–10-week-old pigs [12,101], and vaccine safety was sustained for up to 180 dpi [102]. During the monitoring period, most pigs only exhibited low-level viremia, although viral titers reached up to 10^7^ HAD_50_/mL in some individuals [12,100]. High levels of ASFV-specific antibodies (10^3^–10^5^/mL) were detected starting from 30 dpi and persisted through to 180 dpi [102]. Under laboratory conditions, neither virus nor ASFV-specific antibodies were detected in direct-contact pigs up to 28 dpi, indicating minimal transmission potential of ASFV-G-ΔI177L [12,100,101]. However, under field conditions, approximately 50% (5/10) of contact pigs developed ASFV-specific antibodies, reflecting a limited but detectable risk of virus transmission [101]. Intramuscular vaccination conferred effective protection through low doses (10^2^HAD_50_), and high doses (10^6^ HAD_50_), which induced similar antibody responses. The challenge virus showed minimal replication in vaccinated animals, although sporadic persistence of viral genomes in the tonsils and spleen was detected at 49 dpi (21 dpc), highlighting the potential for limited tissue-level viral persistence despite clinical protection [12]. The protective mechanism of ASFV-ΔI177L has not yet been fully elucidated; however, available data indicate that this vaccine induces a milder inflammatory response and that there is a strong correlation between ASFV-specific antibody responses and the level of protection [12,99,100]. 

Notably, ASFV-G-ΔI177L has also demonstrated high protective efficacy against highly virulent ASFV strains currently circulating in Vietnam, with a minimum protective dose of 10^2^ HAD_50_ providing complete protection in both European commercial pigs and domestic Vietnamese pigs four weeks after vaccination [101]. While ASFV-G-ΔI177L induced full protection against the homologous genotype II strain ASFV-G and partial protection (80%) against Biotype 1 (Ghana2021), it did not protect against heterologous Biotypes 3–6 (Pret4, Uganda65, Kenya1033, and Malawi) [11]. Although ASFV-Georgia2010 and Pret4 share identical p72 sequences, vaccination failed to protect against Pret4. This finding underscores that p72-based analysis alone does not reliably predict cross-protective immunity, and that ASFV classification should be based on whole-genome analyses together with biological characteristics (biotyping). In this context, recent studies have developed combined gene-deleted vaccine candidates based on the *I177L* backbone, including ASFV-G-ΔI177L/ΔMGF110-5L-6L [103], ASFV-GZΔI177LΔCD2vΔMGF [104], and ASFV-G-ΔI177L/ΔEP402R [105]. These recombinant viruses retain high protective efficacy without inducing clinical signs, highlighting their potential as safer and more effective DIVA-compatible ASFV vaccines.

### 5.2. ASFV-G-ΔI177L/ΔLVR

To improve replication efficiency in continuous cell lines, ASFV-G-ΔI177L/ΔLVR was developed and demonstrated to retain a similar level of attenuation and protective efficacy while exhibiting enhanced replication capacity in the PIPEC cell line, thereby highlighting its potential suitability for large-scale vaccine production [95]. ASFV-G-ΔI177L/ΔLVR harbors deletions of *I177L* and nine additional genes (10,842 bp) within the left variable region (LVR) of the ASFV genome [95]. This vaccine candidate demonstrated a high safety profile and conferred effective homologous protection in challenge studies, even at low inoculation doses, with ASFV-specific antibodies maintained at consistently high levels for approximately two months post-vaccination [96]. In experiments on both sows and fattening pigs, viremia after vaccination reached a peak at 7–10 days post-inoculation (dpi), then declined gradually and stabilized until the end of the experiment (including after challenge). Vaccinated pigs maintained good body conditions [95,96,97]. Pregnant sows in late gestation (90–95 days) were vaccinated with ASFV-G-ΔI177L/ΔLVR at a dose of 10^3.5^ TCID_50_. The results demonstrated that the ASFV-G-ΔI177L/ΔLVR strain did not affect reproductive performance in sows, and they developed a robust antibody response. ASFV-specific antibodies were detected at 7–11 days post-vaccination and showed an increasing or stable trend up to 56 dpi [96]. A strong antibody response was induced in vaccinated sows and antibodies were transferred passively to piglets via colostrum. Piglets that ingested colostrum from vaccinated sows within 48 h exhibited seropositivity for ASFV-specific antibodies. During the post-vaccination monitoring period, viral shedding via the oral cavity and feces was detected, but only at low levels [97]. Consistent with previous vaccine evaluation studies, vaccinated pigs maintained a normal health status. The most prominent clinical sign observed was transient fever, even when animals were vaccinated at a dose of 10^6^ HAD_50_ [95,96,97]. Pregnant sows vaccinated during late gestation showed no clinical signs, including fever [97]. Pigs were challenged with a highly virulent ASFV genotype II strain at 28 days post-vaccination, and 100% of the vaccinated animals survived the challenge experiments. The pigs maintained normal health status following challenge, with transient fever being the most prominent clinical sign observed [95,96]. The safety and protective efficacy of ASFV-G-ΔI177L/ΔLVR were comparable to those of the injectable vaccine candidates ASFV-G-ΔI177L and ASFV-G-ΔMGF. 

### 5.3. ASFV-G-ΔMGF

LAV constructs that target deletions in *MGF* genes have shown promise in achieving complete attenuation of viral virulence, thereby improving both vaccine candidates’ safety and protective efficacy. The replication of MGF-deleted strains in PAMs or BMDMs varies substantially depending upon the parental strain. ASFV-G-ΔMGF, derived from the ASFV-G parental strain, retained efficient replication [14], while ASFV-Δ9L [106] and ASFV-Δ9L/Δ7R [107], which originate from the ASFV CN/GS/2018 strain, exhibited markedly reduced replication. In addition, the extent of attenuation, levels of viremia, viral shedding through secretions, and protective efficacy differed among studies [13,14,44,51,107,108,109,110]. These discrepancies may be attributable to differences in the parental virus selected for gene deletion, cell line culture, routes and doses of inoculation, or the experimental pigs’ genetic characteristics.

ASFV-G-ΔMGF is generally safe and does not induce overt clinical disease following challenge with a highly virulent strain, and is associated with low levels of viremia and a strong antibody response. Animals vaccinated with ASFV-ΔMGF at doses ranging from 10^2^ to 10^5^ HAD_50_ exhibited few to no clinical signs for at least 21 days post-vaccination, during which, only a few animals showed transient mild fevers (40.2–40.5 °C) [44], indicating complete attenuation of ASFV following deletion of six MGF genes [13,14,44,111]. Viremia may or may not be detected after ASFV-ΔMGF vaccination; when present, it is either transient [44] or persists at low levels, and in all cases, it declines gradually over time [13,14,111].

Following vaccination with ASFV-G-ΔMGF, ASFV-specific antibodies were detected in 2/5 and 4/5 animals at 14 days post-vaccination (dpv), and in 3/5 and 5/5 animals at 21 dpv at doses of 10^4^ and 10^3^ HAD_50_, respectively. Antibody levels were subsequently maintained, and a booster dose at the same level administered after 21 dpv was required to increase antibody titers further, which peaked at the time of virulent challenge (42 dpv) [44]. In contrast, ASFV-G-ΔMGF-DMAC elicited stronger humoral immune protection, with antibodies detected in 8/10 animals at 14 dpv and in 10/10 animals by 21 dpv; antibody titers peaked at 28 dpv and were sustained over an extended period, including after virulent challenge [111].

ASFV-G-ΔMGF-DMAC has a gene deletion profile similar to ASFV-G-ΔMGF, but was passaged serially 30 times in Diep’s macrophage (DMAC) cells, which resulted in increased stability and attenuation [111]. ASFV-specific antibodies appeared at 14–21 days post-vaccination (dpv), peaked at 42 dpv, and were detected in 100% of pigs by 21 dpv, remaining at high levels after challenge. Following challenge with a highly virulent ASFV strain, vaccinated pigs showed 100% protection, with only mild transient reactions. Post-challenge viremia was detected at low levels at 4 days post-challenge with 10^3^ HAD_50_ and declined to undetectable levels by 28 days post-challenge (dpc). Protection was complete when challenge occurred at 28–150 dpv, but decreased to 40–60% when challenge was performed at 14 or 180 dpv, indicating that the optimal duration of protective immunity remains to be determined [111].

Studies using the Benin97/1 (genotype I) and Georgia2007/1 (genotype II) strains to delete and/or inactivate up to 10 MGF genes have also reported divergent outcomes [51,110]. The Benin-ΔMGF strain replicated efficiently in PAMs, showed marked attenuation, and conferred 66–100% protection depending upon dose and route of inoculation [51]. In contrast, Georgia-ΔMGF exhibited reduced replication in porcine bone marrow cells and protected only 25% of pigs after two immunizations [110]. These findings indicate that, although they target similar MGF regions, protective efficacy is strongly influenced by the viral genetic background. In addition to gene-deleted variants, several MGF-based attenuated vaccines have been generated using alternative approaches. ASFV-989, derived from heat-attenuated ASFV Georgia 2007 isolated from infected animals, induced clinical signs in 17% (3/17) of pigs but provided effective protection against field virus challenge [108]. ASFV Arm07ΔMGF, obtained after 20 passages of ASFV Arm07 in Immortalized Porcine Kidney Macrophages (IPKMs), exhibited a clearly attenuated phenotype; however, only 67% (8/12) of pigs survived challenge with the parental strain, with prolonged fever and persistent viremia observed [109].

## 6. Challenges in Applying Live Attenuated Vaccines

Whether attenuation is achieved through natural selection or artificial biotechnological approaches, electing an appropriate disease prevention strategy using LAVs poses substantial challenges, particularly for a virus such as ASFV, which is characterized by high virulence and limited genetic stability. Key concerns include vaccine virus shedding following administration, viral carryover across pig batches within long-term farm populations, the risk of reversion to virulence, potential vertical and horizontal transmission, host immune pressure on circulating field strains, and the potential for genetic recombination between vaccine strains and field viruses. In addition, continued genetic diversification of LAV strains may alter their virulence phenotypes further. These challenges have been addressed explicitly in the draft guidelines of the World Organization for Animal Health (WOAH), which emphasize that the development and evaluation of ASF vaccine candidates must adhere to stringent validation criteria before regulatory approval for field use.

### 6.1. Genetic Stability and Reversion to Virulence

Genetic stability represents a critical criterion for live attenuated ASF vaccines as these strains may accumulate mutations, continue to evolve, revert toward their parental genotypes, assemble deleted genomic regions, or undergo recombination with circulating field viruses, thereby posing an unpredictable risk of virulence reversion. Intergenotypic recombination has been clearly demonstrated under both in vitro and in vivo conditions, indicating that ASFV is capable of generating genetically chimeric viruses through gene complementation between distinct genotypes [112]. 

Antigenic diversity that arises from recombinant strains represents a major challenge to the development of LAVs. In practical applications of vaccines for RNA viruses, numerous cases of recombination between field and vaccine strains have resulted in the emergence of novel viral variants. This scenario poses two critical challenges; first, newly generated recombinant strains may inherit antigenic features from field viruses or vaccine strains, or display novel antigenic profiles distinct from their parental viruses. Such antigenic alterations complicate the differentiation between field and vaccine strains, particularly when shared antigenic characteristics exist, which significantly hinders outbreak investigation and control efforts. A notable example of this phenomenon is porcine reproductive and respiratory syndrome (PRRS) in China. Second, recombination between vaccine and field strains may lead to enhanced virulence. Although rare, such events can cause vaccinated animals to develop unexpectedly severe clinical disease or facilitate the dissemination of newly emerged, more pathogenic viral strains within pig populations [113].

Reversion to virulence represents the most significant limitation of LAVs. The attenuated phenotype of ASFV-G-ΔI177L was reported to remain stable after five consecutive passages in pigs, despite the presence of minor genomic variations [16]. However, another study demonstrated that following three to four serial passages in pigs, ASFV-G-ΔI177L exhibited partial reversion to virulence, with acute clinical signs observed and mutations identified in the *C257L* gene [19]. These different findings reflect phenotypic stability’s strong dependence upon culture conditions and the genetic background of the parental virus, indicating that ASFV-ΔI177L’s stability requires more rigorous and context-specific evaluation, particularly under field-relevant conditions characterized by multiple interacting factors. Notably, in vivo conditions, where ASFV preferentially targets specific host cell populations, may reveal underlying instability more clearly. Further, the pre-existing presence of co-infecting pathogens in vaccinated pigs, particularly immunosuppressive agents such as PRRSV and PCV2, may create synergistic or antagonistic interactions that influence viral evolution and the potential for virulence reversion.

The use of LAVs in pregnant sows represents a significant safety concern. While the HLJ/18-7GD vaccine has been reported to be safe in pregnant sows [13], ASFV-G-ΔI177L has been shown to induce prolonged viremia (>35 dpi), a high stillbirth rate (43%), and transplacental viral transmission, with only 17% of piglets surviving to 25 days of age [19]. These findings demonstrate that the virus is able to cross the placental barrier, thereby posing substantial challenges to reproductive performance safety when considering large-scale vaccine deployment. In addition, recent field reports from Vietnam have documented the emergence of ASFV-G-ΔMGF–like viruses associated with reproductive disorders in commercial sow herds [18], which further reinforces concerns for potential spillover or partial to complete reversion to virulence under natural conditions. Collectively, the evidence available indicates that the protective efficacy of MGF-deleted ASF vaccines ranges from approximately 60% to 100%, depending upon the parental strain, vaccine dose, route of administration, and the experimental pigs’ genetics [44,108,111]. However, several concerns remain:

(i) Incomplete attenuation, as sporadic cases of mortality or chronic disease have been reported in vaccinated pigs;
(ii) Persistent low-level viral load in tissues such as the spleen, lymph nodes, lungs, and tonsils, which may create a risk of recombination or reversion to virulence [13,17];
(iii) Heterogeneous protective efficacy across different genotypes or biotypes, particularly between genotype I and genotype II strains [82];
(iv) Strong influence of field conditions on vaccine safety and efficacy, including poor health status of vaccinated pigs attributable to pre-existing diseases, infections that occur before or after vaccination, and stresses commonly found in large-scale production systems.

### 6.2. Vaccine Efficacy Against Emerging Variants

Vaccine efficacy against emerging ASFV variants has become a topic of increasing concern as the virus continues to evolve and spread globally. This growing diversity poses a major challenge for vaccine development, particularly with respect to achieving cross-protection among different genotypes and field-circulating variants. LAV strains such as BA71ΔCD2, ASFV-G-ΔI177L, and mutants that target MGF360/505 genes have demonstrated high levels of protection against homologous challenge and partial cross-protection against certain heterologous genotype II strains circulating in Asia and Eastern Europe [12,114]. However, protective outcomes have been inconsistent across studies and appear to depend upon the challenge strain used, the degree of genetic difference, and experimental conditions. Notably, several recently emerging variants harbor alterations in genes involved in interferon antagonism or surface antigen expression, which may reduce vaccine-induced protection or shorten the duration of immunity [82,85].

The emergence of novel ASFV variants, including recombinant strains between genotype I and genotype II reported in several countries, as well as variants that involve the loss or acquisition of MGF genes, raises significant concerns for potential immune escape from currently deployed vaccines [85]. Consequently, vaccine efficacy depends not only upon nature vaccine properties, but also upon robust surveillance strategies and continuous updating of molecular epidemiological data. Although two major vaccine strains, ASFV-G-ΔI177L and ASFV-G-ΔMGF, have been licensed commercially in Vietnam, laboratory-based studies indicate that they do not provide complete protection against genotype I/II recombinant strains, particularly when such variants harbor mutations in immune-regulatory genes [82,85]. Moreover, limited data are currently available with respect to the protective performance of existing ASF vaccines against other emerging genotype II variants circulating in the field, which underscores the need for further experimental and field-based evaluations.

### 6.3. Safety and Bio-Surveillance

Deployment of LAVs requires a high degree of caution and strict biosecurity surveillance programs, including regular and stringent monitoring of vaccine virus circulation, together with the implementation of DIVA (Differentiating Infected from Vaccinated Animals) strategies to ensure reliable discrimination between vaccinated pigs and those infected with field viruses. LAV candidates showed good protection but maintained the ability to replicate in the facility, raising concerns about the risk of vaccine virus dispersal into the environment. In a study by Kitamura et al., 2025 [115], the *S273R* gene-deleted virulent ASFV was found to be safe in vaccinated pigs and simultaneously reduced the ability to replicate in the facility. Although the desired level of protection (30%) could not be achieved, this result lays the groundwork for future studies on optimized ASFV vaccines. In addition, incorporation of molecular markers into LAV strains is essential for tracing their source, thereby ensuring biosafety during vaccine deployment and minimizing the risk of persistence and dissemination of live vaccine viruses within production systems.

To enhance replication in continuous cell lines and facilitate industrial-scale production, several research groups have developed modified ASFV strains, including ASFV-G-ΔI177L/ΔLVR, ASFV-G-ΔI177L/ΔMGF110-5L-6L, ASFV-GZΔI177LΔCD2vΔMG -F, ASFV-G-ΔI177L/ΔEP402R, and ASFV-G-Δ9GL/ΔUK/ΔEP153R [96,103,104,105]. These strains retain attenuated phenotypes and protective immunogenicity while also allowing for the development of genetically engineered DIVA vaccines, consistent with global ASF control strategies. Importantly, demonstration and assurance of vaccine safety through genetic stability are critical, which requires not only proof of in vitro stability but also sustained in vivo stability under diverse field-relevant environmental pressures over extended periods. Such rigorous evaluation is essential to mitigating the long-term risk of generating unforeseen pathogenic variants “X” due to the circulation of insufficiently validated live vaccine strains as part of short-term disease control strategies.

## 7. Prospects for ASFV Vaccines in Vietnam

The prospects for the development and application of ASF vaccines in Vietnam are considered one of the most significant advances since the disease’s emergence in 2019. As ASF continues to cause substantial losses to the swine industry, the identification of a feasible vaccine solution represents not only a technical breakthrough, but also a pathway toward more effective and sustainable disease control strategies. Vietnam has emerged as a regional and global pioneer in the field by implementing field trials of LAVs derived from the ASFV Georgia strain with targeted deletions of key virulence-associated genes, including NAVET-ASFVAC^®^ (*I177L* gene deletion), AVAC ASF LIVE^®^ (deletion of six *MGF360/505* genes) [116], and DACOVAC ASF2 (deletion of *I177L* gene and 9 genes in LVR) [95]. This milestone reflects a strategic shift from passive control measures based upon eradication and veterinary quarantine toward proactive disease management grounded in herd immunity.

Based upon ASFV’s biological characteristics and observations from field outbreaks on small-scale, biosecurity-high-level farms in Vietnam, the basic reproduction number (R_0_) of ASF is relatively low and is estimated to range from approximately 1.01 to 2.32 [117]. The herd immunity threshold (1 − 1/R_0_) varies depending on the production scale, epidemiological characteristics, and transmission dynamics of the pathogen. Under certain conditions, when approximately 50% of the herd is immune, pathogen transmission may be substantially limited. Accordingly, a vaccination-based strategy designed to induce herd immunity, in combination with strict biosecurity measures, is considered a highly feasible approach for ASF control. Nevertheless, the selection and deployment of ASF vaccine candidates must prioritize a high level of safety to avoid the well-recognized limitations associated with LAVs, as discussed in detail in the section “Challenges in Applying Live Attenuated Vaccines.”

In the coming years, the prospects for ASFV vaccines in Vietnam will depend largely upon the successful development and rigorous evaluation of next-generation LAV candidates, particularly gene-deleted strains with demonstrated genetic stability and no risk of reversion to virulence. Marker (DIVA) vaccines are expected to enable reliable differentiation between naturally infected and vaccinated pigs within surveillance programs, thereby facilitating disease management, quarantine enforcement, and animal movement control [118].

However, these prospects can be realized only if complementary measures are implemented in a coordinated manner. Vietnam must strengthen biosecurity systems across both large-scale and smallholder farms further, as vaccine effectiveness under field conditions is related closely to the level of viral exposure. In parallel, the legal framework and technical guidelines governing the use of LAVs require further refinement, including standards for vaccine production, post-vaccination evaluation, vaccine virus surveillance, and more advanced regulatory provisions for LAV biosafety. Close coordination between scientific research, governmental management, and on-farm practices will be pivotal to the success of future ASFV vaccination programs. Given its position as a pioneer in vaccine field trials and post-vaccination viral genetic surveillance, Vietnam is well placed not only to achieve effective domestic ASF control, but also to contribute valuable lessons and strategic approaches for global ASF management, potentially serving as a reference model for countries facing widespread and region-specific ASF challenges in Asia.

This review provides a comprehensive and up-to-date synthesis of ASFV genomic evolution in the context of vaccine development, with a particular focus on Vietnam as a real-world model of post-licensure vaccine deployment. By integrating peer-reviewed studies with national surveillance data and field observations, the review offers a translational perspective that bridges molecular virology and practical disease control. However, several limitations should be acknowledged. First, as a narrative structured review, the study does not include a quantitative meta-analysis, which may limit the ability to statistically compare findings across studies. Second, the heterogeneity of study designs, viral strains, and experimental conditions may introduce variability in data interpretation. Third, some recent findings, particularly those from national surveillance reports or conference proceedings, may not yet be fully peer-reviewed. Finally, the rapidly evolving nature of ASFV, especially the emergence of recombinant strains, means that conclusions may require continuous updating as new data become available.

## 8. Conclusions and Recommendations

The rapid evolution and dynamic shifts in molecular characteristics have contributed to substantial molecular epidemiological diversity within the ASFV population in Vietnam, which underscores the need for comprehensive surveillance of both viral genotypes and phenotypes as part of ASF control strategies. In this context, a critical decision must be made with respect to long-term approaches—whether to pursue complete eradication of ASFV or adopt short-term control through large-scale vaccination designed to establish population-level immunity. In practice, biosecurity remains the most widely implemented control measure among pig producers, with substantial improvements in biosecurity standards observed across farms nationwide following successive ASFV incursions. Nevertheless, a high proportion of farms remain hesitant to adopt currently available ASF vaccines. LAVs continue to represent a promising option; however, their deployment is contingent upon stringent prerequisites for both safety and high protective efficacy.

## Data Availability

Not applicable.
